# The Complexity of Being a Parent in the Hospital and a Patient at Home: A Qualitative Study on Parenting Concerns and Challenges Among Parents With Cancer

**DOI:** 10.1097/NCC.0000000000001276

**Published:** 2023-09-21

**Authors:** Maria Romare Strandh, Emma Hovén, Renita Sörensdotter, Karin Stålberg, Pia Enebrink, Lisa Ljungman, Anna Wikman

**Affiliations:** Author Affiliations: Department of Women’s and Children’s Health (Ms Romare Strandh, and Drs Hovén, Stålberg, Ljungman, and Wikman), Centre for Women’s Mental Health during the Reproductive Lifespan (WOMHER) (Ms Romare Strandh and Dr Wikman), and Centre for Gender Research (Dr Sörensdotter), Uppsala University; and Department of Clinical Neuroscience, Karolinska Institutet (Dr Enebrink), Sweden.

**Keywords:** Cancer, Family, Family support, Neoplasms, Parenting, Parents, Psychosocial intervention, Psychosocial support systems, Social support

## Abstract

**Background:**

Parents given a diagnosis of cancer must balance the demands of their illness and caregiving responsibilities. This can result in parental stress and have a negative impact on the well-being of the whole family. A greater understanding of the experiences of parents with cancer is necessary to provide adequate support.

**Objective:**

The aim of this study was to explore parenting concerns and challenges among parents with cancer who were caring for dependent children younger than 18 years.

**Methods:**

Semistructured interviews were carried out with 22 parents with cancer. Interviews were audio-recorded, transcribed, and analyzed using thematic analysis.

**Results:**

Parental concerns and challenges affected parents in their parental role and their everyday family life. Three overarching themes described the struggles in balancing life as a parent and as a patient: *navigating dual roles as a parent with cancer*, *impact of cancer on parenting*, and *impact on family life*. Parents’ primary focus was on their children’s well-being, and they struggled to manage their own expectations of parenting and the demands on their role in the family.

**Conclusion:**

The results highlight the complexity of being a parent with cancer while caring for dependent children. To support parents during the cancer journey, it is important to understand the consequences of their illness on their parental role and the family.

**Implications for Practice:**

Supporting parents to feel secure in their parental role and providing support to them during their cancer journey should be integrated into routine cancer care, where parenting concerns and challenges are addressed.

Cancer is a growing global burden, with 19.3 million new cases worldwide yearly.^1^ In Sweden, more than 60 000 adults are given a diagnosis of cancer each year.^2^ Between 14% and 25% of adults with cancer are also parents with dependent children (<18 years old).^3^ While parents given a diagnosis of cancer may literally be struggling with life and death, they still have to worry about someone else’s needs, namely, those of their children. Having to balance parenthood and cancer is often physically and emotionally exhausting, because parents need to manage the demands of their illness while also managing caregiving responsibilities. As such, parenthood has been identified as a source of major stress throughout the cancer experience,^4–7^ and single parents face an additional burden compared with those who have a partner.^4,5^ Many previous studies focus on the experiences of mothers with cancer,^6,8–17^ where parenting concerns such as a sense of failure in fulfilling the maternal role, worrying about their children’s well-being more than their own, and difficulties communicating with their children about the illness have been raised.^6,8,12,18,19^ On the other hand, fathers’ parental stress has often been found to be rooted in their inability to work and be the family provider.^19^

Cancer is a huge life stressor, possibly even more so for parents of dependent children, because it has a negative impact on parental psychological well-being, which includes how well parents cope with being a parent, the satisfaction with the role as a parent, and perceived support and general emotional stability.^20^ Poor parental well-being is associated with ineffective parenting strategies and increased parenting problems.^21^ It can also lead to parental stress, or caregiver burden, and arises when the perceived demands on the parental role are greater than the perceived resources available to handle them.^22^ This, in turn, can manifest as depression, anxiety, worry, guilt, and shame, as well as declining family function, which may result in negative consequences within and outside the family.^23^

Several studies have also shown that parental well-being is dependent on children’s well-being and that the relationship is reciprocal.^4,18,24^ Negative adverse effects from the cancer and its treatment can leave parents with a feeling that they are not able to meet their children’s needs, which amplifies the concerns about the impact of cancer on their children.^25^ A cancer diagnosis and its consequences, for example, poor health-related quality of life and psychological distress, negatively impact parenting self-efficacy beliefs and change parenting behaviors, including less interaction with their children when parents feel ill or weak.^6,25^

Still, there is limited knowledge about the experiences, concerns, and challenges of having cancer while parenting dependent children, calling for studies to explore the distress experienced by parents related to their cancer.^4,6^ A review of qualitative studies showed that cancer changes parents’ understanding and fulfillment of the role as a parent and forces them to balance 2 worlds, of being a caregiver and being a patient.^19^ The specifics of this balancing act need to be further investigated, because parents’ well-being is affected by psychosocial and parenting issues, and these struggles need to be prioritized and addressed.^26^ Nevertheless, there is a lack of supporting interventions targeting parents with cancer in routine cancer care. Although a few have been developed, they have not been fully evaluated and implemented.^5,27^ A greater understanding of the complex experiences of parents with cancer is necessary to develop effective support interventions that can be implemented to facilitate the cancer journey for parents.^13^

## AIM

The aim was to explore parenting concerns and challenges among parents with cancer who were caring for dependent children younger than 18 years. This includes experiences in relation to the parent they want to be and their parental role, as well as challenges within the family.

## METHODS

A qualitative design was used to gain an in-depth understanding of parents’ experiences of concerns and challenges related to parenting during their cancer journey. Data were obtained using individual semistructured interviews; an inductive reflexive thematic analysis was used.^28^ To understand parents’ experiences from their subjective point of view and gain a broad insight into the life of parents with cancer, a social constructionist inquiry (ie, our understanding of the world is not fixed but socially constructed) with a hermeneutic phenomenological perspective (ie, knowledge about lived experiences and their meaning are constructed through interpretation) guided the research.^29–31^ Social constructivism, and hermeneutic phenomenology, emphasizes situated knowledge, meaning that knowledge is not objective or universal but is situated within specific social, cultural, and historical contexts.

This study was approved by the Swedish Ethical Review Authority (Dnr 2021-02642). The integrity of the participants and the data were protected in accordance with the Public Access to Information and Secrecy Act (SFS 2009:400) and the General Data Protection Regulation.

## PARTICIPANTS AND PROCEDURE

Adults given a diagnosis of any type of cancer who were also a caregiver to at least 1 child younger than 18 years were eligible for this study. Exclusion criteria included having finished cancer treatment more than 5 years ago and not being able to complete the interview in Swedish or English. Participants were recruited between August 2021 and February 2022 using advertisements distributed by 21 patient organizations, a project website, social media (eg, Facebook groups/pages/ads and well-established Instagram profiles), and information sheets placed at oncology clinics in Sweden. The advertisement contained information about the study and contact details for the research team. In addition, snowball sampling was used, where participants spread the information about the study to other parents whom they knew had cancer. Interested parents contacted the research team by email and received more information about the study, its procedures, and the conditions for participating. Complete information for participants was sent via email or post, together with a consent form that was returned to the research team when signed. Written informed consent was obtained from all participants, and transcripts and presented results were fully anonymized to maintain confidentiality.

Thirty eligible parents were interested in participating, of which 8 did not respond after the initial contact and 1 reminder. The remaining 22 parents were included and interviewed. The interviews conducted were considered sufficient to provide a variety of experiences from parents with cancer in which patterns of concerns and challenges could be identified.^32,33^

## DATA COLLECTION

Interviews were conducted between September 2021 and March 2022 via video link (Zoom), telephone, or face-to-face, depending on participants’ preferences. The same member of the research team (M.R.S.) performed all interviews. Interviews lasted between 29 and 99 minutes (average length, 64 minutes).

The interviewer followed an interview guide with open-ended questions to enhance context-rich descriptions relating to 4 topics: *mental well-being*, *parenthood*, *family life*, and *relationship to partner* (if in a partnered relationship). The interview guide was developed from the previous literature, but the interviews were not limited to these topics, allowing participants to talk freely and share their experiences, with the possibility for the interviewer to ask follow-up questions for more details or clarification.^33^ Examples of the questions on the topics were “How has cancer impacted your parenting?”, “What has been most challenging being a parent with cancer?”, and “Can you describe the communication within your family before and after the diagnosis?” At the start of the interview, participants provided background information (eg, age, gender, family constellation, and cancer diagnosis) both to provide context to their experiences and to build trust between the participant and the interviewer.

## DATA ANALYSIS

Interviews were recorded and transcribed verbatim by a professional transcriber. To understand the lived experiences of the parents, Braun and Clarke’s^28,34^ reflexive thematic analysis was used, which is an analysis method that draws on phenomenological concepts and principles to guide the analysis of qualitative data.^33^ Thematic analysis is reflexive, meaning that no researcher can set aside their preconceptions, and encourages researchers to provide the context of their interpretations and the studied experiences.^35–37^ The transcripts were analyzed in 6 steps: (1) familiarization, (2) identify codes, (3) generate themes, (4) review themes, (5) define themes, and (6) report the results. After these steps, 2 authors (M.R.S. and E.H.), experienced in conducting qualitative research, familiarized themselves with the data by reading and rereading the transcripts, as well as listening to the audio recordings when necessary. The same 2 authors then, separately, carried out an inductive coding to identify relevant parts of the data related to parenting concerns and challenges. Codes were compared and discussed until consensus was achieved and grouped together to generate themes and subthemes that described parents’ experiences. The conceptualization of the themes was an active, dynamic process, and transcripts and codes were revisited multiple times by the same authors (M.R.S. and E.H.) with input from 3 of the other authors (A.W., L.L., and R.S.) and a group of 6 parents with cancer as research partners, before final definitions were formed.

## RESULTS

### Sample

Twenty-two parents, 18 mothers and 4 fathers aged between 27 and 53 years, were interviewed. The parents were all Swedish born and cisgendered, and had a wide range of cancer diagnoses (eg, breast cancer, bile duct cancer, endocrine cancer, and lung cancer), and most (68%) had 2 dependent children living at home. Five participants were single parents, and the rest were in a heterosexual partnered relationship with the children’s other parent or another person who was also a caregiver. In describing parents, partnered parents are mentioned as mothers or father (because of being in the majority in this study), and single parents are described as single mothers or single fathers. Ten parents described that they were living with incurable cancer at the time of the interview. A summary of the characteristics of the study participants is shown in Table 1.

**TABLE 1:** Study Participants (N = 22)

Characteristic	n (%)
Age, y	
Mean (range)	42.09 (27–53)
Gender	
Female	18 (82)
Male	4 (18)
Civil status^a^	
Cohabitant	17 (77)
Length of relationship, y	
Mean (range)	17.23 (3.5–32)
Single parent	5 (23)
Cancer diagnosis	
Bile duct cancer	2 (9)
Blood cancer	2 (9)
Brain cancer	1 (5)
Breast cancer	4 (18)
Cervical cancer	1 (5)
Colorectal cancer	2 (9)
Endocrine cancer	2 (9)
Head and neck cancer	1 (5)
Lung cancer	3 (13)
Melanoma	1 (5)
Sarcoma	2 (9)
Thyroid cancer	1 (5)
Incurable cancer	10 (45)
Cancer treatment status	
Receiving treatment: curative	4 (18)
Receiving treatment: chronic	9 (41)
Not receiving treatment: remission	8 (36)
Not receiving treatment: palliative care	1 (5)
No. children	
Total no. children	39
Mean per parent (range)	1.77 (1–3)
Age of children^b^	
Infant	1 (3)
Toddler	1 (3)
Preschooler	8 (20)
School-aged child	11 (28)
Adolescent	18 (46)

^a^All participants who were cohabitant were in a heterosexual relationship. All single parents had separated from the children’s other parent, or the other parent had died.

^b^Children’s ages are displayed in age spans: infant (ages 1 month to 1 year), toddler (ages 1-2 years), preschooler (ages 2-6 years), school-aged child (ages 6-12 years), and adolescent (ages 12-18 years).

### Interview Analyses

The analysis of the interviews resulted in 3 overarching themes and 10 subthemes (see the Figure). The first 2 themes describe concerns and challenges to the parental role and to themselves as a parent, namely, *navigating dual roles as a parent with cancer* and *impact of cancer on parenting*, whereas the last theme describes challenges within the family, *impact on family life*. Examples from the analysis process are shown in Table 2.

**FIGURE:**
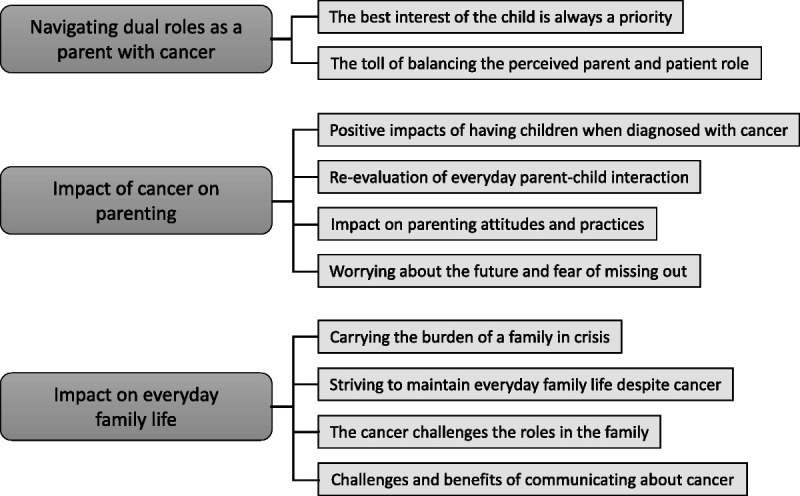
Overview of themes.

**TABLE 2:** Example of the Analysis Process

Theme	Subtheme	Code	Quotes
Navigating dual roles as a parent with cancer	The best interest of the child is always a priority	The child(ren) in focus all the time	“...and then it was like, well I just had a bit of cancer, and it sort of was placed in the background. Because I had to focus on the children the whole time, so it was like something I did on the side, in a way.” (Single mother with curable cancer and 1 toddler and 1 school-aged child)
		Don’t want to burden the children	“I don’t want them to go around being scared all the time that I will die. Like ‘oh this might be the last time,’ the last summer…the last Christmas. No, I have to keep those thoughts to myself.” (Mother with incurable cancer and 1 school-aged child and 2 adolescents)
Impact of cancer on parenting	Worrying about the future and fear of missing out	Equip the children for a life without mum/dad	“I feel stressed about that I have so much I want to pass on to her [the child] before I disappear from her life.” (Mother with incurable cancer and 2 adolescents)
		Fear of dying and leaving their children behind	“And like, leaving the children, that was my first thought, that I can’t leave the children. Like…it was my biggest fear there and then, that the children would be left alone.” (Single mother with curable cancer and 1 preschooler and 1 school-aged child)
Impact on everyday family life	Striving to maintain everyday family life despite cancer	Try to live despite being sick and not let cancer affect everyday life	“We want a normal life as much as possible despite my illness. It shouldn’t be an excuse or something to adapt to. I don’t want to be treated differently in any way because of this [cancer].” (Father with incurable cancer and 1 adolescent)
		How we show intimacy is unchanged	“He [the child] sleeps with us again. We have returned to that routine so…no, nothing has changed. We have been able to go back to how things were before and how I wanted it to be.” (Mother with curable cancer and an infant)

### Theme 1: Navigating Dual Roles as a Parent With Cancer

In this theme, parents expressed that they saw themselves as parents first. Their children’s well-being was the number 1 priority, and parenting expectations often remained the same after diagnosis, which made it hard to balance the role of being a parent and a cancer patient. This theme comprises 2 subthemes: (*a*) *the best interest of the child is always a priority*, and (*b*) *the toll of balancing the perceived parent and patient role*.

#### The Best Interest of the Child is Always a Priority

Parents, regardless of cancer type and state of disease, described that they always kept their children’s best interest at the back of their minds, despite their own well-being. One single father with incurable cancer and 1 adolescent explained: “I don’t think that the cancer is about me; it’s about her [the child]. [...] it’s not me I feel the most sorry for, for having this disease; it’s she who suffers the most.” Parents felt they needed to sacrifice their own well-being to spend time with and take care of their children. These sacrifices were often active decisions to put their children first. Parents protected their children from the negative consequences of having cancer, for example, by withholding expressing negative emotions or poor well-being. They did not want to burden their children and were fearful of hurting their children, for example, scaring them, by showing them how sick they really were. Consequently, parents felt alone in their struggles, because they had to keep many of their thoughts and emotions to themselves.

#### The Toll of Balancing the Perceived Parent and Patient Role

Having cancer sometimes made it impossible to take care of one’s children due to the adverse effects of cancer and the demands on the patient role, for example, sudden trips to the hospital, not being able to breastfeed because of radioactivity from scans or treatments, or fear of catching a cold from the children before surgery. However, the expectations that parents had on parenting and who they wanted to be as a parent often stayed the same, even after parenting conditions changed after the cancer diagnosis. Multiple challenges, with 1 distinct challenge of being fatigued, made it hard to fulfill these parenting expectations. For example, parents described difficulties in meeting children’s needs, both emotional and practical, because of their own struggles. There was a constant worry among parents about if they were caring for their children in the best way. One single mother with incurable cancer and 2 adolescents explained: “I don’t know if I will receive chemotherapy; if I do, I won’t be able to be that super mum that I want to be, and that bothers me a lot.” Adverse effects from cancer also took a toll on parents’ patience with their children, and parents expressed feeling guilty when they lost their temper or could not be emotionally available when their children needed it. Overall, parents felt guilt and shame when they failed to meet their expectations on parenting.

### Theme 2: Impact of Cancer on Parenting

Cancer impacted parenting in many ways. Parents described also positive impacts of the experience of having a child when diagnosed with cancer and that parenting was valued higher than before the cancer. However, parents had concerns about how their cancer would, or had, affect/ed their parenting and their opportunity to be there for their children as the disease progressed. These concerns and consequences of their disease are described in 4 subthemes: (*a*) *positive impacts of having children when diagnosed with cancer*, (*b*) *re-evaluation of everyday parent-children interaction*, (*c*) *impact on parenting attitudes and practices*, and (*d*) *worrying about the future and fear of missing out*.

#### Positive Impacts of Having Children When Given a Diagnosis of Cancer

Although having children during the cancer journey made it complex, parents expressed that it gave a higher sense of security, and gave them strength to go through treatments and try to stick to everyday routines. This helped parents to focus on the healthy aspects of life, which improved their psychological well-being, and made sure that the cancer diagnosis and all the negative consequences did not consume them. One single mother with a preschooler and a school-aged child described it as follows:

You can’t let it [the cancer] consume you and that’s why I think that I don’t suffer so much from depression right now. The children […] pull you back to here and now. They need to be picked up and dropped off, this and that. So you return to everyday life, whether you want to or not.

#### Reevaluation of Everyday Parent-Child Interaction

Parents believed that they had become better parents after their cancer diagnosis, because they felt more available and more present when they spent time with their children. They also described that they appreciated being a parent more. Insights such as life is fragile and short were a revelation parents faced after the cancer, and everyday parenting became treasured. The time with their children became more important and to be cherished. Moreover, parents described a greater need to be close to their children, a need the children seemed to share. A mother with incurable cancer and 2 preschoolers said:

It’s positive [to have cancer] because I cherish every moment I get. I enjoy the hugs more. Putting the children to bed previously felt like a hassle, but now it’s my favorite time of the day, even though it might take 45 minutes.

#### Impact on Parenting Attitudes and Practices

Parents described that their parenting style, including parenting attitudes and practices, had changed since they received their cancer diagnosis. For example, they prioritized parenting responsibilities differently and, as a result, avoided conflicts and set fewer firm boundaries. It became more important to nurture a good relationship with the children, rather than disciplining them. Changes to the parental role were often not perceived as active choices, rather inescapable circumstances, but were still hard to accept. A mother with incurable cancer and 2 preschoolers explained these challenges:

I have a hard time saying no and to set boundaries and that kind of stuff […] and I know it is stupid because it benefits them, but I just want, you know, quality time. I don’t want to fight and such. It’s really, really hard.

#### Worrying About the Future and Fear of Missing Out

Stress and concerns about the future and uncertainty of the cancer disease were common. Parents expressed grief of possibly not having a chance to live a long life as a parent and not being able to follow their children as they grew up. This included a fear of missing out on meaningful life experiences such as graduations, weddings, and possible future grandchildren. One of the greatest fears shared by parents was of dying and leaving their children behind, which they were not ready to do. Some also felt that they had no one who could provide sufficient support for their children if they died, whereas parents with strong social support felt safe that others would take care of their children. As pictured by a mother with incurable cancer and 2 preschoolers:

I will die from cancer if nothing else happens before that. I’m worried and anxious, not about death, but that I won’t be there for my children. […] They are too young, and I won’t have enough time […] and it’s the heaviest burden to bear, and I can’t solve it. I can’t write manuals for my husband for each year about what he needs to do.

The parents raised concerns about how their children would cope if they died and described a constant worry that the cancer disease or death would become a trauma in their children’s lives. To tackle this worry, parents tried to prepare their children for a life without them, both practically and emotionally. One way to prepare their children for their possible death was to “rush” parenting to give their children the tools they believed their children needed in adult life. “Rushing” parenting included transferring their worldview upon their children, teaching the children practical things such as paying bills, and trying to experience as many things as possible with their children.

### Theme 3: Impact on Everyday Family Life

This theme describes how cancer shaped everyday family life, something the parents emphasized the cancer did in many ways. Children’s and partners’ well-being and needs changed, family roles were challenged, family life needed to be adapted yet remained the same, and communication about cancer became a sometimes difficult but central part of the family life. This is described in 4 subthemes: (*a*) *carrying the burden of a family in crisis*, (*b*) *the cancer challenges the roles in the family*, (*c*) *striving to maintain everyday family life*, and (*d*) *challenges and benefits of communicating about cancer*.

#### Carrying the Burden of a Family in Crisis

Children reacted differently to their parent having cancer, and their psychological well-being often declined. The parents described behavioral changes in their children, with acting out, treating them differently, and increased worry being the most noticeable. Simultaneously, as the whole family was in crisis, the sick parent still had to handle the family’s changed well-being. One mother with a preschoolers and a school-aged child described her struggles:

I wasn’t allowed to have anxiety. […] I had a colleague who had breast cancer and she could sit on her sofa with a cup of tea, rest and be sad, but I wasn’t able to do that. There was no place for that because it was snacks, dinner, things to handle, the kids needed to be dropped off and picked up, and more.

One way in which parents tried to support their children’s psychological well-being was to include them in their cancer journey and treatments, such as bringing them to the hospital to help them understand what they had to go through. Parents described a sense of security to know that the children understood what it meant to have cancer.

#### The Cancer Challenges the Roles in the Family

The cancer sometimes leads to changes in the family dynamic. Parents described that family roles were renegotiated, often because of lack of energy to fulfill the parenting role as before. The changed family roles were described as necessary but also sometimes hard to accept, especially if the changed roles remained after the sick parent felt better or recovered. One mother with a preschooler and a school-aged child described that her husband took a greater responsibility when she was sick and became the primary attachment figure, instead of her, which was one of the hardest things to handle.

In other cases, family roles were maintained, despite the cancer illness. Difficulties arose when family roles were too rigid, although the parent felt that they needed to change for their own well-being. For example, many mothers, who often described themselves as “project leaders” in the family, experienced that their partner did not step up when they needed them too and the burden of the responsibility of keeping the family together still depended on their own efforts. One mother of 2 adolescents explained:

[I] fix, plan, organize, make sure there’s food, that the grocery shopping has been done. I plan ahead with things, while [my partner] thinks everything will sort itself out. Well, yes, it will because [I] will sort it out, […]. So, I would have wished that he had taken more responsibility those days [when I felt poorly]. And I mean, it was… three days for each treatment round.

#### Striving to Maintain Everyday Family Life Despite Cancer

Being sick with cancer contributed to a lack of energy and poor well-being, which placed limitations on how parents could interact and spend time with their family, for example, in joining family activities. Parents expressed that their energy levels and stamina could fluctuate from day to day and were hard to predict. Some families were not as affected, or not affected at all. Expectations on what family life should look like played a part in how parents perceived these changes.

Regardless of the parents’ limitations on their well-being, parents described that the family tried to maintain everyday family life as it was before cancer. A strategy used to achieve this was to not let cancer become a big part of the family, which included focusing on the present, not talking about cancer, or sticking to the same family routines as before. Support was recognized as important to maintain everyday family life, both practically and emotionally, from various sources, including extended family, friends, healthcare services, and other community services. Sometimes the perceived need for support was met, but parents felt frustrated when it was not.

#### Challenges and Benefits of Communicating About Cancer

Many aspects of communicating about cancer were challenging for parents, and they described having difficulties in talking to their children in a child-friendly way. This included conveying the seriousness of the disease without scaring the children and the use of age-appropriate language. Parents were careful not to disclose too much detail, which may upset the children or diminish their hope that their parent would be healthy again. Topics that were especially hard to talk about were prognosis and death. Although it was challenging to talk to the children, parents described positive experiences from doing so, and the children seemed to have needed and appreciated it.

Outside the family, parents felt a strong need to be open about their illness, for themselves and for their children. One mother of 2 adolescents clearly stated: “What I felt pretty quickly was that I, like, wanted to be open with this in every way possible. I didn’t want it to be any shame in having a sick parent.” In addition, openness about the cancer had resulted in contact and support from other people with cancer in similar situations, which, in turn, had helped the parents feel less alone in their cancer journey.

## DISCUSSION

This study focused on parenting concerns and challenges among parents with cancer caring for dependent children younger than 18 years. From the experiences shared by the parents in the interviews, which was analyzed using a phenomenological approach to understand the parents’ subjective reality of living with cancer, it was found that concerns and challenges affected both parents themselves in their parental role and their everyday family life. Three themes and 10 subthemes were conceptualized, which described the struggles of balancing life as a parent and a patient.

Congruent with previous studies, the potential and actual impact of cancer on the children was a major source of distress, and parents felt at ease if they knew that their children were doing well.^6,26,38^ Hence, in this study, parental well-being, not surprisingly, was also dependent on children’s well-being, which has been described in the literature on parental cancer.^4,18,24,25^ It seems to be a necessity to make sure that the children are okay for the parents to be okay, and vice versa.

The identity of being a mother or a father was important to parents, but the expectations they had on parenting were hard to fulfill when sick with cancer. It has previously been shown that expectations of parenting during cancer, often from the perspective of the mother’s role, are hard to live up to.^6,18^ Striving to be a “perfect” parent is hard for all parents, but to have cancer and suffer from its adverse effects makes it a lot harder. When parents in this study failed to fulfill their own expectations on being a mother or a father, they felt guilty. This can be seen as one aspect of parental stress,^22^ indicating that there is a relationship between expectations on parenting, inability to fulfill these because of the cancer, and increased parental stress in cancer patients with dependent children. The expectations of parenting and the demands of being a patient can be interpreted as impossible to merge with the resources available to parents.

Changes in parenting behavior have been pointed out in a previous study as one explanation for how parental well-being is affected by cancer, both negatively and positively.^25^ In this study, negative changes were also present and resulted in frustration and sadness. Other changes were described as positive and made parents cherish parenting, consistent with previous findings on meaning-making processes during the cancer journey.^6^ In addition, in a previous study on parenting well-being and cancer, having children have been described as a source of support for parents, providing positive elements.^17^ Parents in this study described that having children was a positive aspect, although it was complex at the same time.

Previous studies have shown that parental stress often manifests as worry and anxiety,^23^ something that was described by parents in this study as well. One source of worry was the future and fear of not being able to live a full and long life as a parent, which was expressed, especially, among parents with incurable cancer. This grief process before an expected death or loss has been described previously among advanced cancer patients.^39^ However, parents in the current study, who were considered to be in complete remission and disease free, still shared many of these concerns and were fearful of recurrence, but they did not share the same amount of stress to equip their children for a future without them. This indicates the various nuances of parental stress regarding concerns about dying and leaving one’s children behind, regardless of whether you have incurable cancer or not at the time.

Parents highlighted the importance of having other reliable adults in the children’s lives. The burden became greater when the parent felt as if no one could take over the caregiving burden when they were sick or if they were to die, regardless of whether they were in a partnered relationship. In contrast, previous literature has shown that parental stress is higher among single parents.^4,5^ One explanation for this discrepancy might be that the additional burden among single parents in previous studies is not only dependent on whether the parent is in a partnered relationship or not. Instead, what is important may be whether a partner, other parent, or another adult is present in the children’s lives in a way that reassures the sick parent that someone else can manage the parenting demands.

Most of the parents interviewed in this study were mothers, and they described their role in the family as the “project leader,” indicating that they were the ones who made everything run smoothly in their family and, if they did not, the whole family would collapse. Fathers expressed similar concerns and challenges but did not identify themselves as the “project leader.” The struggles faced by the mothers, somewhat, contradict the findings from a study by Billhult and Segesten,^10^ where parents (mothers) were interpreted as handling the situation of maintaining everyday family life well. In addition, findings from other studies show that mothers seem to focus more on children’s emotional well-being and experience more fatigue than women without children during the cancer journey. Fathers with cancer more often experience stress about the inability to work and being the family provider and do not experience any additional burden than men without cancer.^17,19^ This indicates that gendered family roles may play a part in parents’ experiences, although there were no clear differences in the experiences of mothers and fathers in this study. However, at times, fathers described their role in the family as more practical, which can be interpreted as gendered.

Children often want information about cancer from their parents,^40^ and the difficulties in talking about cancer within the family is a frequently described challenge,^4,15,16^ reflected also by the experiences of parents in this study. Although parents found it hard to talk about the cancer, they also experienced benefits when they actually did. Parents who were able to communicate well with their children felt that their children coped better with the parent’s illness. As has been reported in several studies, this suggests that parents need more support to feel confident in talking to their children about cancer, because it benefits the whole family.^41^

This study did not specifically study the characteristics of the parents’ children, but it became clear in the interviews that the age of the children seemed to shape parents’ experiences. Parents of young children (infants, toddlers, and preschoolers) described that it was hard to find time to relax and take care of themselves, and many challenges arose from practical things, such as daily routines. Previous findings suggest that parents of younger children report more stress and fatigue^17^ and difficulties with caregiving,^42^ which, in turn, can increase parental stress and decrease parental well-being. However, in this study, young children were also described as being less affected by their parents’ cancer because they were too young to understand, which was a sense of relief for the parents who worried that their cancer affected their children negatively, indicating lower levels of parental stress. Parents of adolescents experienced different struggles, mainly in talking to their children, especially when the child was quiet and distant, and the parent did not get any response. This may be common for adolescents in general but was experienced as problematic in this context, something that has not been well investigated. Hence, parents with adolescents may need different tools to communicate or other ways to ensure that their children are doing okay.

### Strengths and Limitations

A strength of this study was that several authors were involved in all the steps of this study, including from the design of the study to data analysis and reporting, strengthening the credibility of the results. Using thematic analysis does not necessarily need validation through multiple authors, because what is important is the researcher’s subjective skills.^36^ The authors’ different backgrounds have provided a broad and nuanced view of parents’ experiences of having cancer. The authors came from different fields (eg, behavioral sciences, psychology, social sciences, and medicine), and the research partners had different backgrounds, which was a strength. Different perspectives on, for example, disease, illness, sickness, and parenting were constantly discussed. However, the researchers’ preconceptions might have also limited the analysis and the results from the parents’ experience, because it limits the possibility for interpretation due to the researchers’ view.

The qualitative method chosen for this study and the analysis was well suited to explore the lived experiences of parents with cancer. It helped capture their point of view and perspective on being a parent while having cancer, allowing the authors to interpret these experiences to describe concerns and challenges. Furthermore, the methods used were reported transparently to increase dependability and confirmability to help readers follow the data collection and analysis process. The sample of parents in this study was, however, homogenous and consisted of mainly Swedish-born mothers in a heterosexual partnered relationship, which decreases the representability of the results. A limitation regarding representability was that the recruitment of fathers and other parents was challenging, although we specifically targeted fathers, as well as lesbian, gay, bisexual, transgender, and queer parents, in our advertisements. One explanation for the challenges in recruitment may be that breast cancer is the most common cancer type for women at an age where they are parents of dependent children. Another is that fathers and lesbian, gay, bisexual, transgender, and queer parents need different strategies for recruitment to encourage interest in participating. A sample of more diverse parents (eg, from parents with other genders than cis-female and cis-male, born outside Sweden, or in different family constellations) would have increased the understanding of different perspectives on parenting. Thus, the results of this study might not be representative of other parents with other backgrounds. For example, to be able to identify concerns related to gendered family roles, a wider representation of fathers and parents of other genders needs to be included in future studies.

### Implications for Clinical Practice

The results of this study highlight the need for healthcare services to provide adequate support to patients for them to be a parent at the hospital; in the same token, the home environment needs to provide sufficient support needed for the parent to be a patient at home. Nurses at oncology clinics in Sweden are often the ones who have close contact with cancer patients and coordinate their care. To identify and meet the needs of cancer patients who are also parents, this study provides a better understanding of the concerns and challenges they face. This knowledge can facilitate the appropriate support they need, both from healthcare providers and from their families. As evident from the results and previous studies, support addressing the well-being of the whole family could ease the burden for the sick parent. This includes, for example, providing strategies and skills for all family members to be able to cope with the cancer together as a family. Support is needed, especially in talking about the uncertainty of a prognosis and/or death, regardless of the cancer stage^15,43^ as well as knowing how to balance telling the truth and protecting the children.^10^

Existing support interventions have been shown to be helpful and effective in improving well-being among parents,^44–46^ indicating that support needs can be met if interventions were implemented into routine cancer care. Future interventions also need to map the social support network around the family, because the results of this study show the importance of having other adults to rely on who can take care of their children should the parent die.

## CONCLUSION

This study highlights the complexity of being a parent with cancer while caring for dependent children. Parents’ focus is on their children’s well-being during their cancer journey, and they struggle to manage parenting expectations, as well as family life. Through early identification and effective treatments of parental distress, the health and psychological well-being of the whole family could be improved.^47,48^ Findings from this study therefore suggest future research to adapt a 2-fold perspective: parents’ views of parenting and consequences on their parental role, and the family as a unit, to understand how to best support parents during their cancer journey.
